# Molecular genetics and genomics of abiotic stress responses

**DOI:** 10.3389/fpls.2014.00398

**Published:** 2014-08-21

**Authors:** Rohini Garg, Rajeev K. Varshney, Mukesh Jain

**Affiliations:** ^1^Functional and Applied Genomics Laboratory, National Institute of Plant Genome ResearchNew Delhi, India; ^2^International Crops Research Institute for the Semi-Arid TropicsHyderabad, India; ^3^School of Plant Biology and Institute of Agriculture, The University of Western AustraliaCrawley, WA, Australia

**Keywords:** abiotic stress, molecular genetics, genomics, functional genomics, regulatory networks, genetic diversity

Abiotic stresses are the major causes that limit productivity of crop plants worldwide. Plants have developed intricate machinery to respond and adapt over these adverse environmental conditions both at physiological and molecular levels. Due to increasing abiotic stress constraints, plant biotechnologists and breeders need to devise and employ new approaches to improve abiotic stress tolerance in crop plants. Although the current research has divulged several key genes, gene regulatory networks and quantitative trait loci (QTLs) that mediate plant responses to various abiotic stresses, the comprehensive understanding of this complex trait is still not available. With an objective to understand the plant response/adaptation to various abiotic stresses, a special issue was planned for the journal. The current research topic “Abiotic Stress: Molecular Genetics and Genomics” has a combination of primary research articles, perspective, opinion and review work, written by authorities in their respective fields. These articles provide novel insights and detailed overviews on the current knowledge into different aspects of plant responses and adaptation to abiotic stresses.

The perspective article by Henry (2014) presents genomic strategies for development of climate resilient crop varieties to ensure food security. The discovery of genomic variations and genes associated with climate adaptation found in wild relatives of crop plants via whole-genome resequencing may be directly relevant for implementing breeding approaches to develop environmentally adapted crops. In terms of understanding allelic variations, Roorkiwal et al. (2014) report allele diversity for 10 abiotic stress-responsive genes in the reference set chickpea representing the diversity of global chickpea germplasm. Detailed analysis provides haplotype network as well as estimates on genetic diversity for candidate genes in the germplasm collection. The next article by Deshmukh et al. (2014) highlights the importance of integration of various omics approaches for abiotic stress tolerance in model legume crop, soybean. Significant genomic advances have been made for abiotic stress tolerance in soybean in terms of availability of molecular markers, QTL mapping, genome-wide association studies (GWAS), genomic selection (GS) strategies, and transcriptome profiling. It has been suggested that combining QTL mapping based on GWAS along with transcriptome profiling can provide a valuable approach to identify candidate genes involved in desired trait(s) (Deshmukh et al., 2014). It has been realized that studies in other omics branches like proteomics, metabolomics and ionomics and their integration with genomics are equally important and should be part of future research to understand abiotic stress responses.

Two review articles (Golldack et al., 2014; Nakashima et al., 2014) provide important insights into signaling mechanism and transcriptional regulatory network, and their cross-talk in various abiotic stress responses. Both of these articles highlight the central role of transcription factors (TFs) in abiotic stress response and tolerance mechanisms. Other molecular signaling components, such as mitogen activated protein kinases (MAPKs), reactive oxygen species (ROS) and lipid-derived pathways have also been implicated in plant adaptation to environmental adversity (Golldack et al., 2014). In addition, the crucial role of β-catenin-like armadillo (ARM) proteins in abiotic stress responses has also been anticipated (Sharma et al., 2014). The study of these proteins can provide novel insights into the regulation of abiotic stress responses. Nakashima et al. (2014) suggested that TFs function in crosstalk among various abiotic stress responses and are being utilized to improve abiotic stress tolerance in different crops. However, it is important to examine the molecular effects of overexpression of TFs in addition to stress tolerance, because their overexpression may affect other signaling pathways too. The combing/pyramiding of transgenes for different stresses through molecular breeding can provide superior lines with improved stress tolerance in plants.

Calcium ions play a pivotal role in several signal transduction cascades in plants especially abiotic stress signaling. Calcineurin B-Like proteins (CBLs) function as calcium sensors and modulate the activity of CBL-Interacting Protein Kinases (CIPKs). The CBL-CIPK network helps maintaining proper ion balances during abiotic stresses. The CBL and CIPK homologs are present in all green lineages and phylogenomic analysis suggests their expansion from a single CBL-CIPK pair present in the ancestor of modern plants and algae (Kleist et al., 2014). The conservation of NAF domain and yeast two-hybrid results pointed the presence of physically and functionally connected CBL-CIPK network in plants. It is intriguing to analyze the precise role of CBL-CIPK pairs in abiotic stress responses. Virus-induced gene silencing (VIGS) has emerged as an efficient and robust tool for gene function analysis in plants. Ramegowda et al. (2014) provide an elegant overview of the usage of VIGS in different crop species. The article covers recent advances, limitations and future prospects for characterization of abiotic stress related genes and understanding abiotic stress tolerance mechanism. Sengupta and Majumder (2014) addressed the mechanical-functional trade-off in plant vasculature, which can have an adaptive value under abiotic stress conditions. The authors have also provided physiological and genomic basis of abiotic stress tolerance and new possibilities for bridging physiology and genomics for crop improvement.

In summary, the articles presented here emphasize the involvement of a variety of genes/pathways and regulatory networks in abiotic stress responses. The broad-range of articles involving genomics and breeding approaches deepen our existing knowledge about this complex trait. Further, despite the existing comprehensive knowledge in this area, many questions still remain unaddressed. With the climate change threat, depletion of natural resources and ever increasing global population, sustainable and higher crop production is greatly needed. Therefore, there is an urgent need to employ various approaches and their integration to understand the molecular basis of abiotic stress response and adaptation for the development of stress-tolerant crop varieties.

## Conflict of interest statement

The authors declare that the research was conducted in the absence of any commercial or financial relationships that could be construed as a potential conflict of interest.

## References

[B1] Deshmukh R. Sonah H. Patil G. Chen W. Prince S. Mutava R. (2014). Integrating omic approaches for abiotic stress tolerance in soybean. Front. Plant Sci. 5:244 10.3389/fpls.2014.00244 24917870 PMC4042060

[B2] Golldack D. Li C. Mohan H. Probst N. (2014). Tolerance to drought and salt stress in plants: unraveling the signaling networks. Front. Plant Sci. 5:151 10.3389/fpls.2014.00151 24795738 PMC4001066

[B3] Henry R. J. (2014). Genomics strategies for germplasm characterization and the development of climate resilient crops. Front. Plant Sci. 5:68 10.3389/fpls.2014.00068 24616732 PMC3934019

[B4] Kleist T. J. Spencley A. L. Luan S. (2014). Comparative phylogenomics of the CBL-CIPK calcium-decoding network in the moss *Physcomitrella*, *Arabidopsis*, and other green lineages. Front. Plant Sci. 5:187 10.3389/fpls.2014.00187 24860579 PMC4030171

[B5] Nakashima K. Yamaguchi-Shinozaki K. Shinozaki K. (2014). The transcriptional regulatory network in the drought response and its crosstalk in abiotic stress responses including drought, cold, and heat. Front. Plant Sci. 5:170 10.3389/fpls.2014.00170 24904597 PMC4032904

[B6] Ramegowda V. Mysore K. S. Senthil-Kumar M. (2014). Virus-induced gene silencing is a versatile tool for unraveling the functional relevance of multiple abiotic-stress-responsive genes in crop plants. Front. Plant Sci. 5:323 10.3389/fpls.2014.00323 25071806 PMC4085877

[B7] Roorkiwal M. Nayak S. N. Thudi M. Upadhyaya H. D. Brunel D. Mournet P. (2014). Allele diversity for abiotic stress responsive candidate genes in chickpea reference set using gene based SNP markers. Front. Plant Sci. 5:248 10.3389/fpls.2014.00248 24926299 PMC4046317

[B8] Sengupta S. Majumder A. L. (2014). Physiological and genomic basis of mechanical-functional trade-off in plant vasculature. Front. Plant Sci. 5:224 10.3389/fpls.2014.00224 24904619 PMC4035604

[B9] Sharma M. Pandey A. Pandey G. K. (2014). β-catenin in plants and animals: common players but different pathways. Front. Plant Sci. 5:143 10.3389/fpls.2014.00143 24782881 PMC3989760

